# Mineral Springs

**Published:** 1852-11

**Authors:** 


*Mineral Springs.—Notice.*—Doctor John Bell (Philadelphia,) who is preparing a work on *Mineral Springs*, more especially on those of the United

States, is desirous of procuring, at an early day, all accessible information on the subject. With this view he requests his professional brethren to transmit to him all the facts in their possession which may throw light on the chemical composition and curative powers of the waters of the springs in their respective neighborhoods.

Proprietors of these waters would oblige by sending to Dr. Bell authenticated accounts on these points, and also of the topography of the springs, and the roads by which they are approached.—*Philadelphia Med. Examiner.*

